# Combined magnetic resonance and optical imaging of head and neck tumor xenografts using Gadolinium-labelled phosphorescent polymeric nanomicelles

**DOI:** 10.1186/1758-3284-2-35

**Published:** 2010-11-26

**Authors:** Rajiv Kumar, Tymish Y Ohulchanskyy, Steve G Turowski, Mark E Thompson, Mukund Seshadri, Paras N Prasad

**Affiliations:** 1 Institute for Lasers, Photonics and Biophotonics, SUNY at Buffalo, Buffalo, New York, USA; 2 Department of Pharmacology and Therapeutics, Roswell Park Cancer Institute, Buffalo, New York, USA; 3 Department of Chemistry, University of Southern California, Los Angeles, California, USA; 4 Department of Dentistry and Maxillofacial Prosthetics, Roswell Park Cancer Institute, Buffalo, New York, USA; 5 Department of Physics, Northeastern University, Boston, MA

## Abstract

**Background:**

The overall objective of this study was to develop a nanoparticle formulation for dual modality imaging of head and neck cancer. Here, we report the synthesis and characterization of polymeric phospholipid-based nanomicelles encapsulating near-infrared (NIR) phosphorescent molecules of Pt(II)-tetraphenyltetranaphthoporphyrin [Pt(TPNP)] and surface functionalized with gadolinium [Pt(TPNP)-Gd] for combined magnetic resonance imaging (MRI) and NIR optical imaging applications.

**Methods:**

Dynamic light scattering, electron microscopy, optical spectroscopy and MR relaxometric measurements were performed to characterize the optical and magnetic properties of nanoparticles *in vitro*. Subsequently, *in vivo *imaging experiments were carried out using nude mice bearing primary patient tumor-derived human head and neck squamous cell carcinoma xenografts.

**Results:**

The nanomicelles were ~100 nm in size and stable in aqueous suspension. T1-weighted MRI and relaxation rate (R1 = 1/T1) measurements carried out at 4.7 T revealed enhancement in the tumor immediately post injection with nanomicelles, particularly in the tumor periphery which persisted up to 24 hours post administration. Maximum intensity projections (MIPs) generated from 3D T1-weighted images also demonstrated visible enhancement in contrast within the tumor, liver and blood vessels. NIR optical imaging performed (*in vivo *and *ex vivo*) following completion of MRI at the 24 h time point confirmed tumor localization of the nanoparticles. The large spectral separation between the Pt(TPNP) absorption (~700 nm) and phosphorescence emission (~900 nm) provided a dramatic decrease in the level of background, resulting in high contrast optical (NIR phosphorescence) imaging.

**Conclusions:**

In conclusion, Pt(TPNP)-Gd nanomicelles exhibit a high degree of tumor-avidity and favorable imaging properties that allow for combined MR and optical imaging of head and neck tumors. Further investigation into the potential of Pt(TPNP)-Gd nanomicelles for combined imaging and therapy of cancer is currently underway.

## Background

Head and neck squamous cell carcinomas (HNSCC) constitute a biologically diverse group of neoplasms that vary in their clinical presentation and therapeutic response [1,2]. Diagnostic evaluation of head and neck tumors often involves the use of non-invasive imaging techniques such as computed tomography (CT), magnetic resonance imaging (MRI) and positron emission tomography (PET). However, currently available advanced imaging modalities vary in their limits of sensitivity, resolution and depth profiling. Development of agents that allow imaging of tumors across multiple platforms could be potentially beneficial for diagnostic and therapeutic evaluation of cancer in patients.

In this regard, nanoparticle-based platforms have several distinct advantages that could potentially allow integration of diagnostic and therapeutic applications in oncology [3-5]. First, the ability to incorporate multiple imaging tracers permits concurrent evaluation of the same nanoformulation across imaging platforms [3]. Secondly, nanoparticles exploit tumor physiological characteristics such as the enhanced permeability and retention (EPR) effect, which enables 'passive targeting' to tumor sites [4]. Thirdly, nanoplatforms offer a traceable chassis onto which specific targeting moieties (antibodies, peptides) can be added according to the desired biological application [5]. Finally, nanoparticles can be used as carriers to selectively deliver high doses of multiple therapeutic agents to cancer sites while minimizing delivery to normal tissues [4,5].

**Figure 1:**
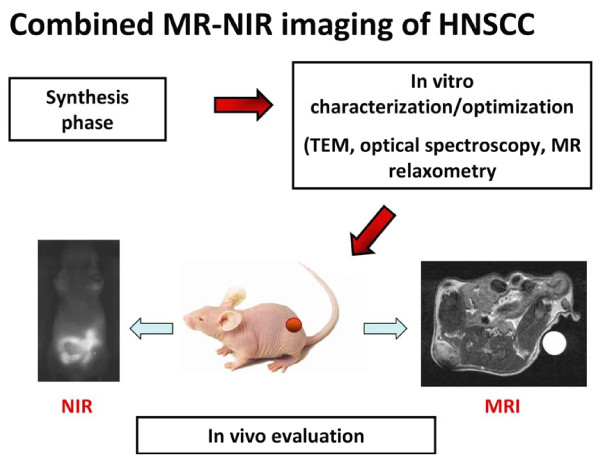
**Schematic of overall study design**. The figure depicts the basic workflow algorithm involved in the design, synthesis and characterization of the nanoparticles for dual modality imaging. Following initial synthesis, characterization of the optical properties of Pt(TPNP)-Gd polymeric nanomicelles was performed using transmission electron microscopy, optical spectroscopy and dynamic light scattering techniques. MR relaxometric measurements were also performed in phantoms and compared to clinically approved MR contrast agents. Subsequently, *in vivo *studies were performed using patient tumor-derived human head and neck squamous cell carcinoma (HNSCC) xenografts to determine the potential of the polymeric nanomicelles for dual modality imaging.

The overall goal of this study was to develop a nanoparticle-based platform for multimodal imaging of head and neck cancer. To achieve this goal, we have developed a phospholipid-based phosphorescent nanomicelle formulation functionalized with gadolinium for combined magnetic resonance imaging (MRI) and optical (near-infrared phosphorescence) imaging of tumors. While the two imaging techniques vary in their resolution, sensitivity and cost of application, it was our hypothesis that development of a contrast agent for both techniques would provide complementary information and enable cross-validation of findings. Here, we report the synthesis and characterization of polymeric phospholipid nanomicelles encapsulating a NIR phosphorescent dye, Pt(II) tetraphenyl-tetranaphthoporphyrin [Pt(TPNP)], and surface functionalized with gadolinium [Gd-Pt(TPNP)] for combined MRI and NIR optical imaging of head and neck tumors. Studies were initially carried out *in vitro *to investigate the optical and MR properties of the polymeric nanomicelles. Subsequently, *in vivo *studies were carried out using patient tumor-derived HNSCC xenografts established in nude mice (Figure 1). The results demonstrate the potential of Gd-Pt(TPNP) nanomicelles as a dual modality imaging agent for molecular imaging of head and neck cancers.

## Methods

### Chemicals

All phospholipids, 1,2-distearoyl-sn-glycero-3-phosphoethanolamine-N-[methoxy(polyethylene glycol)-2000] (DSPE-mPEG-2000), 1,2-distearoyl-sn-glycero-3-phosphoethanolamine-N-[amino(polyethylene glycol)-2000] (DSPE-PEG-2000 NH_2_), 1,2-distearoylglycero-3-phosphocholine (DSPC) and 1,2-dimyristoyl-sn-glycero-3-phosphoethanolamine-N-diethylenetriaminepentaacetic acid (gadolinium salt) (DMPE-Gd) were procured from Avanti Polar Lipids (Alabaster, AL). All other solvents were procured from Sigma-Aldrich, (St. Louis, MO) and were used without any further purification.

### Tumor model system

We have recently described the procedures for establishment of primary patient tumor-derived HNSCC xenografts in SCID mice [6]. Using similar procedures, tumors were established in athymic nude mice. Female athymic nude mice (*nu/nu*, body weight 22-30 g) were obtained from Harlan Sprague Dawley, Inc. (Indianapolis, IN) and housed in microisolator cages on-site at the Roswell Park Cancer Institute's Laboratory Animal Resource. The mice were provided standard chow/water, and maintained on 12-hour light/dark cycles in a HEPA-filtered environment. All procedures were performed in accordance with protocols approved by the Institutional Animal Care and Use Committee at Roswell Park Cancer Institute.

### Synthesis of Pt(TPNP)/Gd nanomicelles

**Figure 2:**
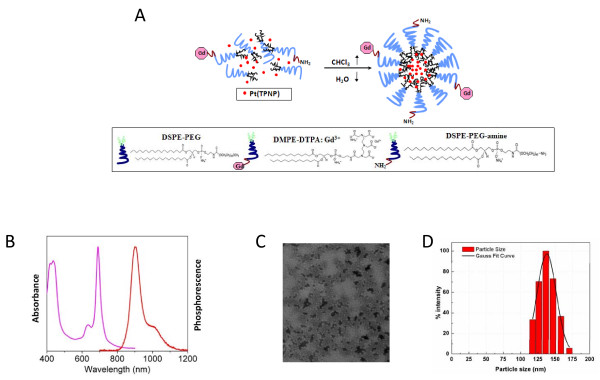
**Synthesis and characterization Pt(TPNP)-Gd nanomicelles**. **(A) **Synthesis of phospholipid nanomicelles encapsulating Pt(TPNP) and surface functionalized with gadolinium, **(B) **absorption and emission spectra of the micelles, **(C) **TEM analysis of the nanoparticles showing a nearly spherical morphology, and, **(D) **dynamic light scattering analysis showing a unimodal distribution of nanoparticle size (103.6 ± 1.8 nm).

We have recently described the procedure for synthesis of nanomicelles encapsulating the phosphorescent dye, Pt(II)-tetraphenyltetranaphthoporphyrin [Pt(TPNP)] in the hydrophobic core of the DSPE-PEG/DSPC nanomicelles [7]. We have modified the reported procedure to incorporate DMPE-Gd into nanomicelles. A schematic describing the encapsulation of the Pt(TPNP) into the DSPE-PEG/DSPC/DMPE-Gd phospholipid micelles is shown in Figure 2. Briefly, 100 μL of phosphorescent dye stock solution in toluene (0.12 mg/ml) was evaporated and dried under vacuum. The obtained solid mass was then resuspended in 1 mL chloroform with 6.33 × 10^-6 ^moles of phospholipids containing 17% of DSPE-mPEG-2000, 17% of DSPE-PEG-2000 NH_2_, 13% of DMPE-Gd and 53% of DSPC. The solution was sonicated for 1 minute and the chloroform allowed to evaporate under vacuum, following which the residue was gently heated at 80°C and 2 mL of water was added to obtain an optically clear suspension containing Pt(TPNP)/DSPE-PEG/DSPC/DMPE-Gd (from here on referred as Pt(TPNP)-Gd nanomicelles). Dye/micelle formulation was purified by ultracentrifugation at 500,000 g for 2 hours. The supernatant was discarded and the pellet containing phosphorescent dye-micelles was resuspended in water. Samples of Pt(TPNP)-Gd nanomicelle formulation were stable in deionized water or phosphate buffered saline (PBS), with no observable aggregation, dissociation or bleaching for at least 1 month of storage. The samples were stored at 4°C for further use.

### Characterization of Pt(TPNP)-Gd nanomicelles

The mean diameter of Pt(TPNP)-Gd polymeric micelles was evaluated by dynamic light scattering (DLS) measurements using a model 90 plus Zeta Sizer (Brookhaven Inc., Brookhaven, NY). Transmission electron microscopy was also performed using a JEOL model JEM-100CX electron microscope operating at an acceleration voltage of 80 kV. To measure the morphology and size distribution of polymeric micelles, a drop of sample solution (1 mg/ml) was placed on a 300-mesh copper grid coated with carbon. Approximately 2 minutes after deposition, the grid was tapped with filter paper to remove surface water and air-dried. Negative staining was performed using a droplet of 2% wt aqueous phospotungistic aciduranyl acetate. The optical absorption measurements were done using a Shimadzu model 3600 UV-Vis-NIR spectrophotometer. A SPEX 270M spectrometer (Jobin Yvon), equipped with an InGaAs TE-cooled photodiode (Electro-Optical Systems, Inc.) was used to obtain the photoluminescence (PL) spectra. A laser diode emitting at 630 nm was used as the excitation source. The sample in a quartz cuvette was placed directly in front of the entrance slit of the spectrometer, and the emission signal was collected at 90° relative to the excitation light.

### Magnetic Resonance Imaging

Magnetic Resonance Imaging (MRI) studies were performed using a 4.7 T/33-cm horizontal bore magnet (GE NMR Instruments, Fremont, CA) incorporating AVANCE digital electronics (Bruker Biospec with ParaVision 3.0.2; Bruker Medical Inc., Billerica, MA) and a removable gradient coil insert (G060, Bruker Medical Inc., Billerica, MA) generating maximum field strength of 950 mT/m and a custom-designed 35-mm RF transmit-receive coil. *In vitro *MR relaxometry was performed in phantoms containing serial dilutions of the nanomicelle formulation over a Gd concentration range of 0-100 μM with varying phospholipid:Gd ratios. T1 relaxation rates (R1) were measured using a Fast Imaging with Steady-State Precession (True-FISP) imaging sequence with the following scan parameters: field of view (FOV) = 3.20 × 3.20 cm, matrix size = 128 × 128, TR/TE_eff_. = 3.0/1.5 ms, NEX = 1, slice thickness = 1.50 mm, TI = 40.0 ms, flip angle = 60° and number of echoes = 60. R1 values were plotted as a function of concentration and linear regression analysis was performed to estimate T1-relaxivities. Estimates were compared to the clinically-approved gadolinium-based contrast agent, gadopentetate dimeglumine (Gd-DTPA; Magnevist^®^).

*In vivo *MRI studies were performed using nude mice bearing subcutaneous primary patient tumor-derived HNSCC xenografts to examine the tumor imaging potential of the nanoparticles. Images were acquired at baseline (before nanoparticle injection), immediately after injection, 4 hours and 24 hours post-administration of (PtTPNP)-Gd nanoparticles (35 μmol Gd/kg). For these studies, animals were anesthetized using the inhalational anesthetic, Isoflurane (Abbott Laboratories, IL), induced at 4% in oxygen, and sustained 2-3% during imaging. The mice were secured in a form-fitted, MR-compatible sled (Dazai Research Instruments, Toronto, Canada) equipped with temperature and respiratory monitoring sensors. The sled, along with a phantom containing 0.15 mM Magnevist, was then positioned inside the scanner using a carrier tube composed of cellulose acetate butyrate plastic (Curbell Plastic, Orchard Park, NY). The animal body temperature was maintained at 37°C throughout the scanning process using an air heater system (SA Instruments Inc., Stony Brook, NY), and temperature feedback was automatically initiated due to thermocouples inherent to the sled, in conjunction with computer software supplied with the heater. Data acquisition consisted of localizer images followed by high resolution axial T2-weighted (T2W) images for anatomic imaging of tumor and normal tissues [FOV = 3.20 × 3.20 cm, matrix size = 256 × 192, TR/TE_eff_. = 2500/41.0, NEX = 4, slice thickness = 1.00 mm, interslice distance = 1.25 mm, and number of echoes = 8]. T1-weighted (T1W) spin echo images were acquired for examining the tumor enhancement pattern at different times post nanoparticle injection [FOV = 3.20 × 3.20 cm, matrix size = 256 × 195, TR/TE_eff_. = 404/7.8 ms, NEX = 4, slice thickness = 1.00 mm, interslice distance = 1.25 mm]. The change in T1-relaxation rate (R1 = 1/T1) of tumor and kidneys was measured using a T1-weighted saturation recovery fast spin echo (T1-FSE) sequence [FOV = 3.20 × 3.20 cm, matrix size = 128 × 96, NEX = 1, slice thickness = 1.00 mm, interslice distance = 1.25 mm, TE_eff _= 25 ms and TR = 6000, 3000, 1500, 750, 500, 360.34 ms]. Finally, MR angiography was performed using a T1/flow-weighted 3D spoiled gradient echo (T1-SPGR) sequence with the following parameters: FOV = 4.80 × 3.20 × 3.20 cm, matrix = 192 × 96 × 96, TR/TE_eff_. = 15.0/3.0, NEX = 1, flip angle = 40° and slice thickness = 32.00 mm. Image processing and analysis were carried out using the medical imaging software ANALYZE (Version 7.0; Biomedical Imaging Resource, Mayo Foundation, Rochester, MN). Regions of interest (ROIs) were manually drawn around tumor, muscle and kidney and object maps were created for calculation of T1 relaxation rates using MATLAB (Mathworks Inc., Version 7.1, Natick, MA).

### In vivo Optical Imaging

Near-infrared optical imaging of tumor-bearing mice was performed at the 24 hour time point following completion of MRI studies. *In vivo *spectral imaging was carried out using the spectral imaging system, Maestro GNIR FLEX comprising of an optical head, an optical coupler and a cooled, scientific-grade monochrome CCD camera, along with image acquisition and analysis software. The polymeric micelles were excited at 650-700 nm using "deep red" excitation filter (CRi), transmitting light from the source (Xe lamp) in the range of 650-700 nm. An emission filter (800 LP) was used to cut off the leaking excitation light. A tunable liquid crystal filter in front of the imaging CCD camera was automatically stepped in 10-nm increments from 800 to 950 nm while the camera captured images at each wavelength with constant exposure. Overall acquisition time was about 10 seconds. The 16 resulting TIFF images were loaded into a single data structure in memory, forming a spectral stack with a spectrum at every pixel. Autofluorescence and the Pt(TPNP)-Gd nanomicelles phosphorescence spectra were obtained from the spectral image using the computer mouse to select appropriate regions. Spectral unmixing algorithms were applied to create the unmixed images of 'pure' autofluorescence and 'pure' phosphorescence signals.

### Statistics

All measured values have been reported as mean ± standard error of the mean. P values less than 0.05 were considered statistically significant. All statistical calculations and analyses were performed using Graph Pad Prism (Version 5.00; Graph Pad, San Diego, CA). A two-tailed paired student's *t *test was used to compare tumor T1 relaxation rates at various time points in comparison to baseline pretreatment values.

## Results

### Synthesis and characterization of (PtTPNP)-Gd nanoparticles

A schematic of the workflow involved in the design, synthesis and characterization (*in vitro *and *in vivo*) of dual modality (PtTPNP)-Gd nanoparticles is shown in Figure 1. Phospholipid nanomicelles encapsulating Pt(TPNP) were synthesized with gadolinium-functionalized surface as shown in Figure 2A. As one can see from the absorption and emission spectra of the micelles presented in Figure 2B, incorporation of DMPE-Gd to the nanomicellar formulation does not significantly affect the basic photophysical properties of Pt(TPNP) encapsulated in the hydrophobic core of the nanomicelles (7), and the Pt(TPNP)-Gd nanomicelles are phosphorescent. TEM analysis revealed a nearly spherical morphology of the nanoparticles with a narrow size distribution (Figure 2C). Zeta potential studies carried out with the Pt(TPNP)-Gd nanomicelles have shown an overall negative zeta potential owing to the presence of the PEG molecules on the surface of the nanomicelles. The net surface charge of the nanomicelles was found to be -38.24 mV. Consistent with the TEM observations, the DLS analysis of the nanoparticles revealed a unimodal distribution with the maximum percentage intensity to be of 103.6 ± 1.8 nm in size (Figure 2D).

### In vivo MR-NIR optical imaging of HNSCC xenografts

**Figure 3:**
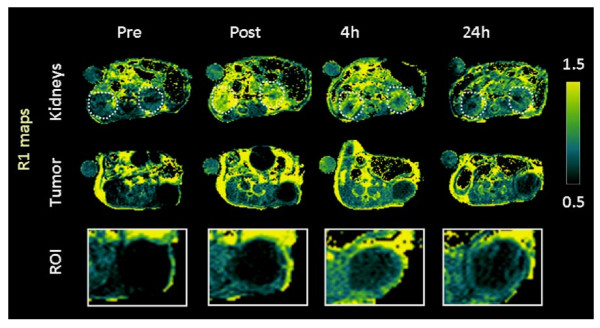
**Magnetic resonance imaging of patient tumor-derived HNSCC xenografts using Pt(TPNP)-Gd nanomicelles**. The panel of images represent T1-relaxation maps calculated before (pre), immediately after (post) and 4 hours and 24 hours post intravenous injection of Pt(TPNP)-Gd nanomicelles. Axial slices of the kidneys (*top panel*, white dotted outline) and the tumor (*middle panel*) are shown. An enlarged view of the tumor is shown in the bottom panel. Signal enhancement was visible immediately after nanoparticle injection and persisted up to 24 hours post administration.

**Figure 4:**
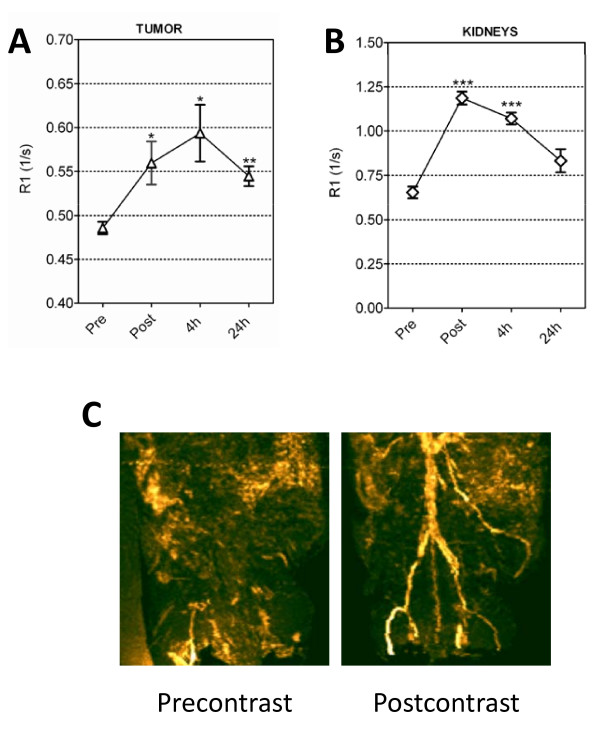
**MR angiography (MRA) and NIR optical imaging of HNSCC xenografts using Pt(TPNP)-Gd nanomicelles**. Measurement of T1 relaxation rates of tumor (A) and mouse kidneys (B) before and at different times after administration of Pt(TPNP)-Gd nanomicelles. A significant increase in R1 was seen in both tumor and kidneys after administration of nanomicelles compared to preinjection estimates. Peak enhancement in the tumor was observed at 4 hours post injection. (C) Maximum intensity projections generated from 3D T1-weighted images acquired post contrast showed visible enhancement in contrast within the tumor, liver and blood vessels.

Prior to *in vivo *investigation, studies were carried out *in vitro *to investigate the MR properties of the gadolinium containing nanomicelles. The T1 relaxivity of Pt(TPNP)-Gd nanomicellar formulation at 4.7T was markedly higher (10.4 mmol^-1^*s^-1^) than the clinically approved contrast agent, Gd-DTPA (3.6 mmol^-1^*s^-1^). Subsequently, i*n vivo *imaging experiments were carried out using nude mice bearing primary patient tumor-derived human HNSCC xenografts. T1-enhancement characteristics and relaxation rate measurements of tumor and blood (kidneys) were performed before (precontrast) as well as 1, 4 and 24 hours after nanoparticle administration (35 μmol Gd/kg). Figure 3 shows colorized T1-relaxation maps (R1 maps) calculated from T1-weighted images of a mouse before (pre), immediately after (post), 4 hours and 24 hours after injection of the Pt(TPNP)-Gd nanomicelles. An axial slice showing the kidneys (*upper panel*) and the tumor (*middle panel*) is shown. An enlarged ROI of the tumor is also shown in the bottom panel. Signal enhancement was visible immediately after nanoparticle injection and persisted up to 24 hours post administration. Measurement of T1 relaxation rates (R1 = 1/T1) showed a significant increase (P < 0.05 at immediately post and 4 h post injection; P < 0.01 at 24 h) in tumor R1 at all 3 time points post nanoparticle administration, compared to preinjection values with peak enhancement in the tumor at 4 hours post injection (Figure 4A). A significant increase in the R1 of the kidneys was also observed immediately after injection and at 4 h post injection (Figure 4B, P < 0.001). Maximum intensity projection (MIP) images generated from 3D T1-weighted images acquired post contrast showed visible enhancement in contrast within the tumor and blood vessels in mice (Figure 4C).

**Figure 5:**
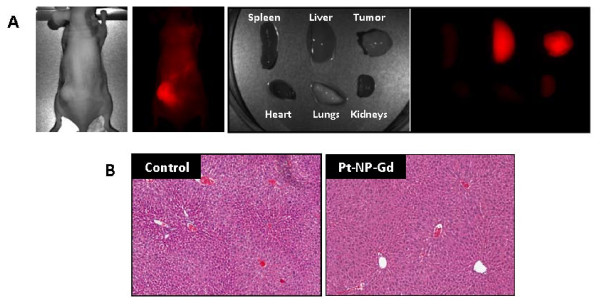
**NIR optical imaging and histologic evaluation of Pt(TPNP)-Gd nanomicelles**. (A) *In vivo *and *ex-vivo *NIR optical images acquired 24 h post administration of Gd-Pt(NPTP) nanomicelles. High contrast phosphorescence signal confirmed tumor localization of the nanoparticles. No phosphorescence signal was detected from other dissected organs (heart, spleen, kidneys). (B) Haematoxylin and eosin stained liver sections of a control animal and an animal injected with Pt(TPNP)Gd nanomicelles (48 hours post injection). No visible change in the histologic architecture of the liver or evidence of necrosis, fibrosis, fatty changes or clear cell changes were seen in treated animals compared to controls.

NIR optical imaging, performed following the completion of MRI at the 24 hour time point, revealed high contrast phosphorescence signal and confirmed tumor localization of the nanoparticles (Figure 5A). The large spectral separation between the Pt(TPNP) absorption (~700 nm) and phosphorescence emission (~900 nm) allowed for a dramatic decrease in the level of background autofluorescence and scattered excitation light. Consistent with previous observations (7), *ex-vivo *NIR optical imaging revealed accumulation of nanoparticles in liver tissues, in addition to tumor localization (Figure 5A). No significant phosphorescence signal was seen from other dissected organs (heart, spleen, kidneys). Histologic evaluation of liver tissues did not show any evidence of toxicity following nanoparticle administration. Figure 5B shows haematoxylin and eosin stained liver section obtained from an animal 48 h following injection of Pt(TPNP)Gd nanomicelles. No visible change in the histologic architecture of the liver or evidence of necrosis, fibrosis, fatty changes or clear cell changes were seen in treated animals compared to controls. Corresponding liver section of an untreated control animal is also shown in Figure 5B.

Toxicologic evaluation of Pt(TPNP) nanoparticles was also performed by measuring the body weight of animals over a 2 week period. Animals injected with Pt(TPNP) formulation underwent physical and neurological evaluations. No changes in eating, drinking, grooming, exploratory behavior, activity, physical features (e.g., weight and skin color), and neurological status were observed in animals injected with the nanoformulation compared to control animals. No significant differences in average body weight were observed between control and treatment groups.

## Discussion

A tremendous amount of scientific effort has been placed in recent years towards the generation of multifunctional nanoparticles for early detection of cancer, targeted drug delivery to tumors and for monitoring of cancer therapy. Nanomedicine has the ability to overcome the limitations of traditional imaging and therapeutic agents and to potentially integrate diagnostic and therapeutic applications in oncology [3-5]. Functionalization of nanocarriers with tumor-targeting moieties provides the ability to selectively deliver anticancer agents at optimal concentrations to tumor sites [5]. In addition, the inclusion of imaging agents enables real time, non-invasive assessment of tumor physiology, drug delivery and therapeutic response [3-5].

The objective of the present study was to develop and characterize a nanoparticle-based platform for dual modality imaging of HNSCC. Here we report the utility of phospholipid-PEG based polymeric nanomicelles encapsulating a NIR phosphorescent dye, and surface conjugated with gadolinium, as an imaging probe for combined MRI and optical imaging. Our results demonstrate that Pt(TPNP)-Gd nanomicelles exhibit a high degree of tumor-avidity and favorable imaging properties that allowed for combined MRI and optical imaging of head and neck tumors.

The polymeric nanoplatform utilized in our study provides several benefits over conventional imaging agents which include prolonged circulation time, improved tumor selective targeting and reduced antigenicity. The nanomicelles are made of biocompatible copolymers and exploit the EPR effect commonly observed in tumors making them ideal carriers for delivery of anticancer drugs, fluorophores and biomolecules selectively to tumors [8,9]. Several preclinical studies have previously demonstrated the usefulness of polymeric micelles as drug carriers [8,9]. A number of polymeric conjugates, micellar and liposomal formulations of drugs are also currently being evaluated in clinical trials [9]. To minimize opsonization, the surface of the nanoparticles was conjugated with polyethylene glycol (PEG), a relatively inert hydrophilic polymer that provides good steric hindrance for preventing protein binding. The net negative charge on the surface of the Pt(TPNP)-Gd nanomicelles should have a pronounced effect on the adsorption of different physiological lipoproteins in systemic circulation, playing a critical role in the clearance of the nanoparticles from the body [10].

The development of a dual modality imaging agent for combined MRI and optical imaging is attractive, given the complementary nature of these two imaging modalities. Optical imaging methods provide a high degree of sensitivity for bioimaging and are relatively inexpensive and easy to use. Traditionally, clinical application of optical imaging has been limited by tissue penetration of visible light. The use of NIR probes for *in vivo *imaging allows for imaging of deeper tissues and reduces the background autofluorescence, providing higher contrast [8,11], but does not fully overcome the drawback of optical imaging related to limited depth of light propagation in biological tissues. In contrast to optical imaging methods, MRI suffers from limited sensitivity and lacks resolution for imaging at the cellular level, but does not have limitation of depth and provides excellent soft tissue contrast. Thus, a nanoplatform that allows for combined MR-NIR imaging could potentially bridge the gaps in sensitivity/resolution and depth of imaging. Indeed, such nanoparticle based approaches for combined MR-optical imaging of tumors has been previously reported [12-14]. These approaches have typically utilized NIR fluorescent dyes, e.g., Cy5.5, in combination with iron oxide or gadolinium for MR contrast. PAMAM-dendrimer based nanoparticles and gadolinium-labeled photoluminescent quantum dots have been examined for their potential for targeted molecular imaging of lymph nodes and tumor vasculature [12-16]. Dual modality MR-optical imaging approaches have also been utilized for sentinel lymph node mapping in mice [16]. Optical tracers have also been studied for diagnostic and therapeutic applications in head and neck cancers [17,18].

We have previously demonstrated that phospholipid-based NIR phosphorescent polymeric nanomicelles exhibit superior optical imaging properties *in vivo *[7]. In the same study, using histologic analyses we have shown that Pt(TPNP) nanomicelles can be safely administered to animals without any associated short-term toxicities. The *in vitro *and *in vivo *results from the present study demonstrate the potential of Gd-labeled phosphorescent nanomicelles as MR contrast agents (Figure 3A, 4A and 4B). Although Gd^3+ ^based agents (Gd-DTPA; Gd-DOTA) have been approved for clinical use and are routinely used in human studies, these agents are associated with considerable limitations. A majority of the clinically approved low molecular weight contrast agents are non-specific in nature and do not contain any targeting moieties. Due to their non-targeted nature, these contrast agents undergo rapid extravasation and plasma clearance (in the order of minutes) resulting in a very narrow imaging window. In this regard, the development of 'targeted' MR contrast agents associated with longer tissue retention times would offer a broader imaging window in which high-resolution images can be acquired. Secondly, the most commonly used Gd-containing compounds have relatively low relaxivities and can be toxic. In particular, at high concentrations of the Gd-containing MRI contrast agents, a significant amount of Gd^3+ ^ions are released and circulate in a free, uncomplexed state. These free Gd^3+ ^ions have been shown to result in severe toxicities leading to nephrogenic systemic fibrosis in patients with compromised renal function [19]. The results of our *in vitro *MR relaxometry studies showed that Gd-containing polymeric nanomicelles exhibit superior MR properties (higher T1 relaxivity) compared to clinically approved Gd-based contrast agents (e.g., Gd-DTPA). The higher relaxivity of Gd-containing nanomicelle formulations could therefore potentially allow reduction in the amount of injected dose of contrast agent, without compromising image contrast. The combination of optical and MR imaging probes also facilitated tracking of the biodistribution and tumor accumulation of the nanoparticles over time.

With increasing interest in the development of novel targeted therapies for management of head and neck cancers, nanomedicine is likely to play a significant role in the diagnostic evaluation, therapy and tumor response assessment to such targeted therapies in patients [20].

## Conclusions

In conclusion, Pt(TPNP)-Gd polymeric nanomicelles reported here serve as efficient probes for dual modality imaging of head and neck tumors in mice. While the mechanisms involved are not entirely clear, the tumor specificity is believed to arise from the combination of the EPR effect and the inherent tumor-avidity of the phosphorescent platinum-porphyrin complex entrapped within the nanomicelles [21]. Further investigation into the potential of Pt(TPNP)-Gd nanomicelles for combined imaging and therapy of head and neck cancers is necessary and is currently underway.

## Competing interests

The authors declare that they have no competing interests.

## Authors' contributions

RK participated in the design of the study, performed the synthesis and characterization of nanoparticles and drafted the manuscript. TYO participated in the design and optical characterization of the nanoparticles and drafting of the manuscript. ST carried out the *in vitro *and *in vivo *MRI experiments, image processing and data analysis and participated in the drafting of the manuscript. MET participated in the design and synthesis of the nanoparticle formulation and reviewed the manuscript for intellectual content. MS participated in the conception and design of the study, MR image acquisition and data analysis, drafting of the manuscript and reviewed the manuscript for critical intellectual content. PNP participated in the conception and design of the study, provided review of optical imaging data and manuscript for critical intellectual content. All authors read and approved the final manuscript.
